# Comparative Analysis of Concurrent Chemoradiotherapy Versus Chemotherapy Alone as First-Line Palliative Treatments for Advanced Esophageal Squamous Cell Carcinoma

**DOI:** 10.3390/jcm13216353

**Published:** 2024-10-23

**Authors:** Jirapat Wonglhow, Panu Wetwittayakhlang, Patrapim Sunpaweravong, Chirawadee Sathitruangsak, Arunee Dechaphunkul

**Affiliations:** 1 Division of Medical Oncology, Department of Internal Medicine, Faculty of Medicine, Prince of Songkla University, Songkhla 90110, Thailand; jirapat.jw@gmail.com (J.W.); spatrapi@medicine.psu.ac.th (P.S.); sjirawadee@gmail.com (C.S.); 2 Division of Gastroenterology and Hepatology, Department of Internal Medicine, Faculty of Medicine, Prince of Songkla University, Songkhla 90110, Thailand; wet.panu@gmail.com

**Keywords:** esophageal cancer, squamous cell carcinoma, advanced stage, chemotherapy, concurrent chemoradiotherapy, efficacy, safety

## Abstract

**Background**: In advanced-stage esophageal squamous cell carcinoma (ESCC), treatment of both the primary tumor and metastatic sites is imperatively required. Consequently, an optimal treatment modality should effectively control both aspects. Therefore, the benefits of concurrent chemoradiotherapy (CCRT) in cases of advanced-stage ESCC should be evaluated. **Methods**: This retrospective study compared the efficacy and safety of CCRT versus chemotherapy alone for advanced-stage ESCC patients from January 2012 to December 2023 at a university hospital in Southern Thailand. Survival was assessed using the Kaplan–Meier approach, with comparisons being made by the log-rank test. A *p*-value of <0.05 indicated statistical significance. **Results**: From a total of 196 patients with stage IV ESCC, 117 (59.7%) received CCRT, while 79 (40.3%) received chemotherapy alone. The median overall survival (OS) time was 9.04 months for CCRT and 5.79 months for chemotherapy (hazard ratio, HR: 0.58 [0.43–0.78]). CCRT significantly improved OS time in stage IVA patients (HR: 0.52 [0.29–0.93]), but not in stage IVB patients (HR: 0.76 [0.51–1.11]). The median progression-free survival (PFS) time was 6.04 months for CCRT and 3.50 months for chemotherapy (HR 0.48 [0.35–0.65]). The objective response rates (ORRs) were 43.6% and 22.8%, respectively (*p* = 0.003). Hematological toxicities were more common with CCRT, along with mild cases of treatment-associated pneumonitis and dermatitis. **Conclusions**: Although palliative chemotherapy is the standard treatment for advanced-stage ESCC, CCRT provides significant benefits for patients with stage IVA ESCC, improving OS, PFS, and ORRs, despite there being a higher incidence of adverse events. Thus, CCRT should be considered for patients with stage IVA ESCC with a good performance status.

## 1. Introduction

Global instances of esophageal cancer reached approximately 604,100 cases in 2020, making it the seventh most prevalent cancer [1]. Despite its prevalence, advancements in treatment have been limited over the past two decades, making esophageal cancer the sixth most common cause of cancer-related deaths globally [1,2,3]. The survival rate for advanced-stage esophageal cancer is notably poor, and is marked by a discouraging five-year survival rate of below 5% [4].

Upon diagnosis, approximately half of patients are ineligible for surgery due to factors such as unresectable disease or medical unfitness [5,6]. For these patients with inoperable or metastatic esophageal cancer, palliative care is the main treatment and is aimed at improving quality of life (QoL) and extending survival. Although advancements in chemotherapy have been made, individuals with metastatic esophageal squamous cell carcinoma (ESCC) still have a median overall survival (OS) time of only 8–11 months with combination chemotherapy [2,3,7,8]. Recent advancements using immunotherapy alongside chemotherapy have shown promise, increasing the OS time to approximately 15 months [3,9,10,11]. However, access to immunotherapy in Thailand remains limited due to high costs.

While radiotherapy (RT) is not typically the first-line treatment for advanced-stage ESCC, it offers supportive care, particularly for managing esophageal bleeding and alleviating obstructions resulting from primary tumors. Concurrent chemoradiotherapy (CCRT) is considered the standard approach for inoperable locally advanced ESCC [12,13,14,15]. However, there is limited information regarding the potential advantages of CCRT for patients with advanced-stage ESCC, including patients with clinical stage T4b and/or M1 ESCC [16,17]. Current guidelines recommend palliative chemotherapy over aggressive RT for primary tumors in patients with advanced-stage ESCC [18,19]. Nevertheless, addressing the primary tumor in cases of advanced-stage ESCC remains crucial, as its progression can lead to debilitating symptoms and affect survival outcomes.

The treatment of advanced-stage ESCC requires the targeting of both the primary tumor and the metastatic lesions. Therefore, an optimal treatment approach must effectively manage both of these aspects. Consequently, this study compared the efficacy and safety aspects of CCRT with those of chemotherapy alone in patients with advanced-stage ESCC.

## 2. Material and Methods

### 2.1. Study Participants and Procedures

We performed a retrospective review of medical records for patients newly diagnosed with ESCC at the Songklanagarind Hospital, Prince of Songkla University, from January 2012 to December 2023. The inclusion criteria were as follows: (1) histologically confirmed ESCC; (2) advanced-stage disease classified based on the 8th edition of the American Joint Committee on Cancer (AJCC) guideline as clinical stage IV (cT4NanyM0, cTanyN3M0, or cTanyNanyM1); (3) patients in receipt of first-line palliative chemotherapy with cisplatin and 5-FU, carboplatin and 5-FU, or carboplatin and paclitaxel, with or without concurrent RT treatment being applied to the primary tumor; and (4) patients aged 18 years or older. The exclusion criteria were as follows: (1) patients who had previously received first-line palliative chemotherapy; (2) patients with another concurrent primary malignancy; and (3) patients who had received concurrent treatment with molecular-targeted therapeutic drugs or immunotherapy.

Patient data were retrieved from electronic medical records through the hospital information system of Songklanagarind hospital. The collected information encompassed initial clinical characteristics including sex, age at diagnosis, body mass index (BMI), smoking and alcohol use, comorbid conditions, Eastern Cooperative Oncology Group (ECOG) performance status, the location of the primary tumor, tumor differentiation based on histopathology, TNM staging, sites of metastasis, baseline laboratory results, and any prior treatments.

The chemotherapy regimens were administered as follows: for the cisplatin and 5-FU regimen, cisplatin was infused at 80 or 100 mg/m^2^ over 1 h on day 1, followed by a continuous 24 h infusion of 5-FU at either 800 mg/m^2^ on days 1–5 or 1000 mg/m^2^ on days 1–4. In the carboplatin and 5-FU regimen, carboplatin was infused at an AUC of 5 over 1 h on day 1, with 5-FU administered similarly to the cisplatin regimen. Both of these regimens were repeated every 4 weeks. For the carboplatin and paclitaxel regimen, carboplatin was given at an AUC of 5 over 1 h on day 1, with paclitaxel administered at 175 mg/m^2^ over 3 h on day 1 in 3-week cycles. All regimens were given for up to four or six cycles or until intolerable side effects, disease progression, death, or a patient’s request to discontinue. Dosage adjustments were made by primary oncologists according to patients’ Eastern Cooperative Oncology Group (ECOG) performance status and baseline laboratory results.

Alternative treatment options are considered if the initial chemotherapeutic regimen is ineffective. The decision regarding subsequent therapy was based on the patient’s performance status, preferences, and accessibility of other available agents.

This study was approved (REC. 67113141) by the Ethics Committee of the Research Center of the Faculty of Medicine, Prince of Songkla University. Given its retrospective design, the necessity for written informed consent was waived. To protect patient confidentiality, all identifying data were omitted from the study.

### 2.2. Measurement

The primary objective of this study was to evaluate OS times between CCRT and chemotherapy alone for advanced-stage ESCC. Secondary objectives included comparison of progression-free survival (PFS), objective response rate (ORR), and safety between the two treatment groups. OS time was measured from the start date of chemotherapy until death from any cause, while PFS time was measured as the time from chemotherapy initiation to either radiologically confirmed disease progression or death, whichever occurred first. To monitor treatment responses, chest and abdominal computed tomography (CT) were performed every 2–3 months. The response rate (RR) was determined following the Response Evaluation Criteria for Solid Tumors (RECIST) version 1.1. Safety was assessed using version 5.0 of the Common Terminology Criteria for Adverse Events to grade adverse events.

### 2.3. Statistical Analysis

Baseline characteristics were reported with continuous variables expressed as either the median with interquartile range (IQR) or as the mean with standard deviation, based on the data distribution. Categorical variables were presented as frequencies and percentages. The Kaplan–Meier method was utilized to generate survival curves, while the log-rank test was employed for comparison. A multivariable Cox proportional hazards analysis was performed to adjust for confounding variables. Statistical analysis was conducted with R software version 3.3.2 (R Foundation, Vienna, Austria). All *p*-values were two-sided, with a *p*-value of <0.05 indicating statistical significance.

## 3. Results

### 3.1. Baseline Characteristics

Overall, 196 patients with advanced-stage ESCC were included in this study; 115 (58.7%) were diagnosed with M1 disease. Among them, 117 (59.7%) received first-line palliative CCRT and 79 (40.3%) received palliative chemotherapy alone. In general, baseline conditions were similar between the two groups, as presented in Table 1. However, patients who received CCRT exhibited a better ECOG performance status, a higher proportion of T4 stage, fewer instances of metastatic disease, fewer organ metastases, and fewer lung and liver metastases. Additionally, they possessed higher baseline hemoglobin and serum albumin levels than those who received chemotherapy alone.

### 3.2. Treatment Information 

The CCRT cohort received cisplatin combined with 5-FU more frequently than those receiving chemotherapy alone. The median number of chemotherapy cycles was four for the CCRT cohort and three for the chemotherapy-alone patients. There was no significant difference in the median doses of carboplatin, cisplatin, 5-FU, and paclitaxel between the two treatment options. Approximately 45% of patients in both groups experienced dose reductions. The median dose of RT administered concurrently with chemotherapy was 50.4 Gy, with 80% of the patients in the CCRT group receiving an RT dose of 50.0 Gy or more. Details of the treatments are listed in Table 2.

In the CCRT cohort, the majority of treatment discontinuations were due to completion of the full treatment regimen, followed by disease progression. Conversely, patients in the chemotherapy alone cohort were more likely to discontinue treatment due to disease progression, followed by death. Approximately 25% of patients in each group underwent subsequent treatment.

### 3.3. OS

**Figure 1:**
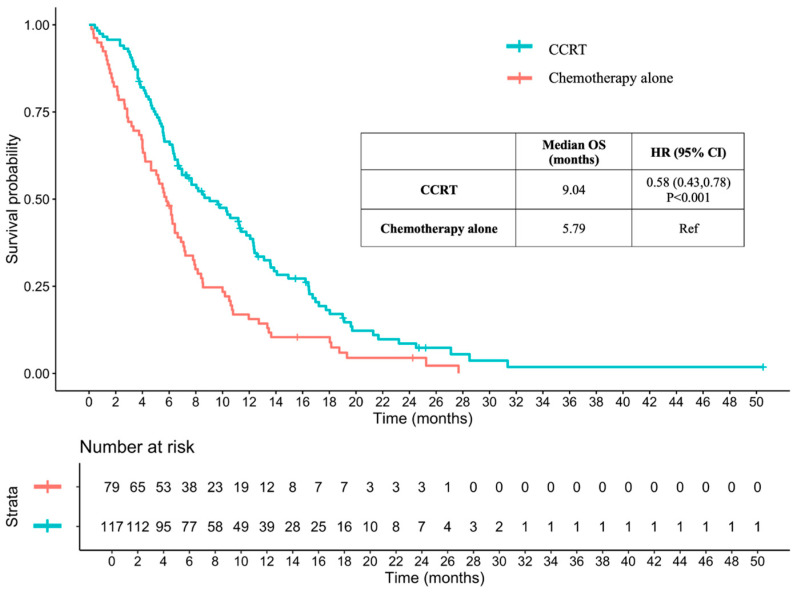
Overall survival time between CCRT and chemotherapy alone in advanced-stage ESCC. OS: overall survival; CCRT: concurrent chemoradiotherapy; HR: hazard ratio; CI: confidence interval; and ESCC: esophageal squamous cell carcinoma.

The median follow-up was 6.68 months. The median OS time for patients receiving CCRT was 9.04 months, compared to 5.79 months for those receiving chemotherapy alone (hazard ratio, HR: 0.58, 95% confidence interval, CI: 0.43–0.78, and *p* < 0.001) (Figure 1). A multivariable Cox proportional hazards model was used to adjust for potential confounding variables, including ECOG performance status, T stage, M stage, lung metastasis, liver metastasis, baseline serum hemoglobin level, and baseline serum albumin levels; the adjusted HR for CCRT compared to chemotherapy alone was 0.80 (95% CI: 0.56–1.16, *p* = 0.248).

**Figure 2:**
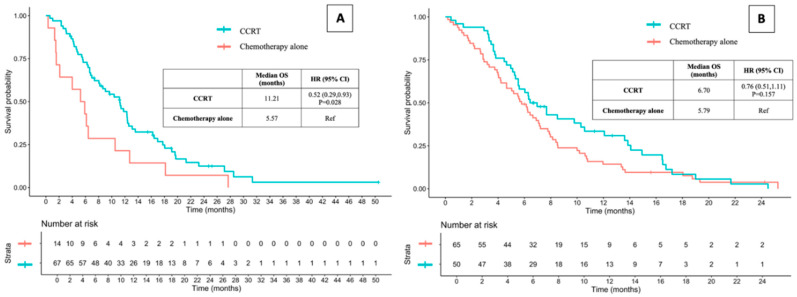
Overall survival time between CCRT and chemotherapy alone in stage IVA (without distant metastasis; (**A**) and IVB (with distant metastasis; (**B**) ESCC. OS: overall survival; CCRT: concurrent chemoradiotherapy; HR: hazard ratio; CI: confidence interval; and ESCC: esophageal squamous cell carcinoma.

**Figure 3:**
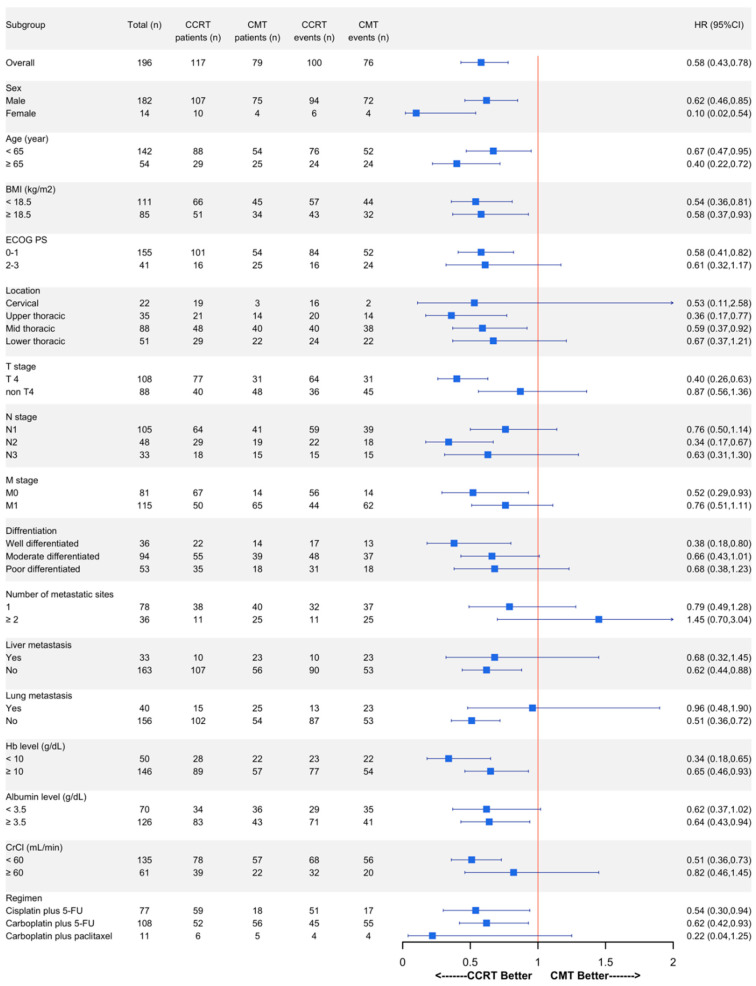
Subgroup analysis for overall survival time between CCRT and chemotherapy alone in advanced-stage ESCC. OS: overall survival; CCRT: concurrent chemoradiotherapy; CMT: chemotherapy; HR: hazard ratio; CI: confidence interval; ESCC: esophageal squamous cell carcinoma; BMI: body mass index; ECOG: Eastern Cooperative Oncology Group; PS: performance status; Hb: hemoglobin; CrCl: creatinine clearance; and 5-FU: 5-fluorouracil.

Considering patients without distant metastasis, the median OS time was significantly increased for those receiving CCRT compared to those receiving chemotherapy alone (11.21 vs. 5.57 months, HR: 0.52, 95% CI: 0.29–0.93, and *p* = 0.028), while there was no statistically significant difference in patients with distant metastasis (6.70 vs. 5.79 months, HR 0.76, 95% CI 0.51–1.11, and *p* = 0.157) (Figure 2). The OS time favored CCRT over chemotherapy alone across various subgroups, as shown in Figure 3, including those categorized by sex, age, BMI, and hemoglobin level. Additionally, CCRT was preferred in patients with good ECOG performance status, T4 stage, M0 stage, absence of lung and liver metastases, no hypoalbuminemia, and those receiving a chemotherapy regimen of cisplatin plus 5-FU or carboplatin plus 5-FU.

### 3.4. PFS

**Figure 4:**
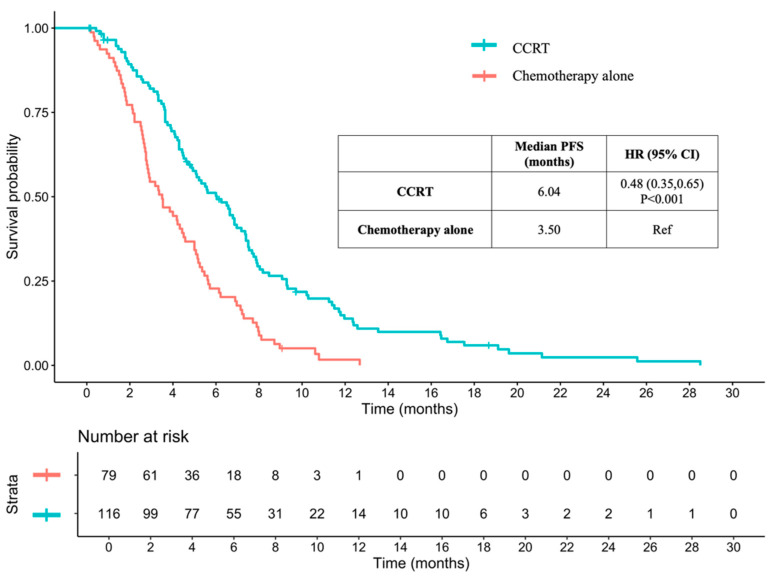
Progression-free survival between CCRT and chemotherapy alone in advanced-stage ESCC. PFS: progression-free survival; CCRT: concurrent chemoradiotherapy; HR: hazard ratio; CI: confidence interval; and ESCC: esophageal squamous cell carcinoma.

**Figure 5:**
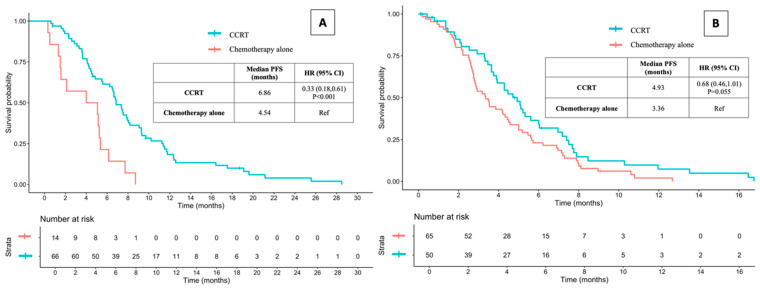
Progression-free survival between CCRT and chemotherapy alone in stage IVA (without distant metastasis; (**A**) and IVB (with distant metastasis; (**B**) ESCC. PFS: progression-free survival; CCRT: concurrent chemoradiotherapy; HR: hazard ratio; CI: confidence interval; and ESCC: esophageal squamous cell carcinoma.

The median PFS time for patients receiving CCRT was 6.04 months, compared to 3.50 months for those receiving chemotherapy alone (HR: 0.48, 95% CI: 0.35–0.65, and *p* < 0.001) (Figure 4). However, the median PFS time was significantly longer for patients without distant metastasis who received CCRT compared to those receiving chemotherapy alone (6.86 vs. 4.54 months, HR 0.33, 95% CI 0.18–0.61, and *p* < 0.001). In contrast, there was no statistically significant difference in median PFS time between patients with distant metastasis who received CCRT and those who received chemotherapy alone (4.93 vs. 3.36 months, HR 0.68, 95% CI 0.46–1.01, and *p* = 0.055) (Figure 5).

### 3.5. ORR

Regarding the RR, as shown in Table 3, the ORR was significantly higher for CCRT (43.6%) than for chemotherapy alone (22.8%; *p* = 0.003). Approximately 20% of the patients did not undergo a response evaluation. However, among those who underwent response assessment, CCRT showed a significantly higher ORR than chemotherapy alone (51.5% vs. 34.0%, *p* = 0.018).

### 3.6. Safety

Table 4 provides a summary of the toxicity profiles. The inclusion of concurrent RT with chemotherapy led to significantly more cases of neutropenia, leukopenia, thrombocytopenia, febrile neutropenia, radiation-associated pneumonitis, and radiation-associated dermatitis. Radiation-associated pneumonitis and dermatitis are mostly mild.

## 4. Discussion

This study compared the real-world efficacy and toxicity of CCRT versus chemotherapy alone as first-line palliative treatments in patients with advanced-stage ESCC. The addition of concurrent RT to chemotherapy improved the OS, PFS, and RR. However, after employing a multivariable Cox proportional hazards model to adjust for potential confounding factors, the benefit of concurrent RT was not observed. These benefits were predominantly observed in patients without distant metastasis (stage IVA) but not in those with distant metastatic disease (stage IVB). It is also important to note that adding RT increased toxicity.

Dysphagia and malnutrition, resulting from primary tumors in esophageal cancer, significantly impact patients’ QoL and survival times [20]. While palliative chemotherapy is essential for controlling systemic disease and improving survival times in patients with advanced-stage ESCC [21], achieving effective local control of the primary tumor is crucial for symptom relief and improving both QoL and survival outcomes. RT is particularly effective in alleviating dysphagia and managing local tumor growth, thereby preventing further tumor invasion and reducing complications. Brachytherapy is another option for relieving dysphagia [22]; however, it is less commonly used and not available in all centers compared to RT. This is due to the need for experienced practitioners and careful patient selection, given the high risk of TE fistula.

Adding palliative RT treatment for patients with well-controlled metastatic esophageal cancer following palliative chemotherapy has been shown to improve PFS time compared to palliative chemotherapy alone [23,24]. However, some patients may experience tumor progression during chemotherapy, potentially missing the opportunity to undergo RT. Consequently, CCRT has been investigated as a treatment option for advanced-stage ESCC. Currently, no guidelines recommend CCRT for advanced-stage ESCC due to insufficient evidence [18,25]. Palliative chemotherapy, with or without immunotherapy, is currently suggested for advanced-stage ESCC, including unresectable T4 or distant metastasis [9,11,18,25].

Our study showed that patients treated with chemotherapy alone had a median OS time of 5.79 months and a median PFS time of 3.5 months. Compared to recent randomized controlled trials (RCTs) of advanced-stage ESCC [9,11], our study showed lower median OS and PFS. The median OS times for patients in the Keynote-590 [11] and Checkmate-648 [9] trials were 9.8 and 10.7 months, respectively, while the median PFS times were 5.8 and 5.6 months, respectively. These differences may be attributed to variations in the patients’ backgrounds between RCTs and real-world settings. Our study included a greater proportion of frail patients with more comorbidities, such as chronic kidney disease, as well as poorer ECOG performance status. Additionally, although our study included patients receiving chemotherapy regimens with either cisplatin or carboplatin combinations, the RCTs included only cisplatin combinations. However, carboplatin is a viable option for cisplatin-ineligible advanced-stage ESCC patients, with comparable OS times, PFS times, and ORRs [21]. This suggests that the RCTs excluded patients who were deemed ineligible for cisplatin, potentially due to poor ECOG performance status or impaired renal function. Furthermore, only 26% of our study participants received subsequent treatments, compared with 60% in the RCTs, which contributed to the lower OS times. Nonetheless, our findings align with previous real-world studies reporting median OS times of 6–7 months [16,26] and a median PFS time of approximately 4 months [16,27]. Collectively, our findings emphasize the differences in OS times related to varying baseline clinical characteristics and subsequent treatments.

Access to immunotherapy in Thailand is limited due to its high cost. Therefore, RT is considered a viable option for enhancing the efficacy of chemotherapy alone. In our study, CCRT substantially increased OS time and PFS time in patients with advanced-stage ESCC compared to those treated with chemotherapy alone. However, due to the retrospective design, some factors were imbalanced between the two groups, potentially confounding survival outcomes. Patients in the CCRT group had better ECOG performance status, fewer distant metastasis, fewer liver and lung metastases, and higher hemoglobin and serum albumin levels, which may have contributed to longer survival outcomes. Additionally, patients in the CCRT group had a higher T stage, possibly indicating symptoms requiring local treatment, leading to selection bias. To account for these factors, we used a multivariable Cox proportional hazards model. After adjustment, CCRT did not significantly improve OS compared to chemotherapy alone.

Subgroup analysis from our study showed that the survival benefit was not observed in patients with distant metastasis (stage IVB). These findings align with those of a prior study, which indicated that CCRT using cisplatin and paclitaxel did not lead to a significant improvement in OS times when compared to chemotherapy alone in metastatic ESCC (16.8 vs. 14.8 months; *p* = 0.56) [28]. Conversely, some studies have demonstrated the benefits of CCRT even in patients with distant metastases [17,29,30]. Differences in patient and tumor characteristics and treatment conditions likely influenced survival outcomes. One Western retrospective study by Guttman et al. (2017) demonstrated a survival benefit of CCRT over chemotherapy alone in metastatic esophageal carcinoma with an OS time of 11.3 vs. 8.3 months (*p* < 0.001) [29]. However, it predominantly included patients with esophageal adenocarcinoma, which comprises approximately 75% of all patients. Another eastern retrospective study by Lyu et al. (2018) showed significantly improved OS times with CCRT compared to chemotherapy alone, at 14.0 vs. 11.0 months (*p* = 0.007) [17]. Although that study included only patients with ESCC, only those with a good ECOG performance status were included, and only cisplatin combinations were allowed with RT doses higher than 50 Gy. Thus, the benefits of CCRT in metastatic ESCC remain inconclusive. Further prospective studies in metastatic ESCC are necessary due to the inconsistencies in outcomes and the limited number of studies.

Our study demonstrated that patients with stage IVA ESCC, an advanced stage without distant metastasis, achieved a survival benefit in terms of both OS time and PFS time from CCRT compared to chemotherapy alone. These findings are consistent with those of an earlier study [16] that reported an OS time of 10.5 vs. 7.6 months (*p* = 0.0029) and a PFS time of 8.9 vs. 5.4 months (*p* = 0.0098). The OS time in our study also aligns with previous single-arm retrospective studies that reported an OS time of approximately 10–12 months [31,32,33]. Taken together, these results suggest that CCRT should be considered for advanced-stage ESCC, especially for stage IVA.

In addition to M staging, subgroup analysis showed that the benefits of CCRT on OS were consistent regardless of sex, age, BMI, or baseline hemoglobin level. Patients with good ECOG performance status, T4 lesion and no hypoalbuminemia derived greater benefit from CCRT over that from chemotherapy alone. T4 lesions require local control to prevent further invasion and complications [34]. Tracheoesophageal (TE) fistula is a potentially serious risk factor for patients receiving CCRT due to its poor prognosis. This event is highly predicted in patients with T4 ulcerative-type tumors and invasion of the bronchus or trachea [33]. Although our study showed that the risk of TE fistula was not significantly higher in the CCRT cohort compared to the chemotherapy alone cohort, this risk should still be considered. Furthermore, the subgroup analysis should be interpreted with caution because the small sample size might have affected the outcomes.

The ORR of palliative chemotherapy alone in our study was consistent with previous studies (approximately 30%) for combination chemotherapy [4,35]. Our study revealed that CCRT improved ORRs by approximately 50%. Tumor response is strongly associated with improved OS [28] and may also improve dysphagia and QoL. However, our retrospective study did not collect QoL data, which should be evaluated in future research, including QoL and dysphagia scores or swallowing improvements. Taken together, although the survival benefit of CCRT remains uncertain, symptomatic primary tumors could be a key factor in considering RT to increase RR and reduce the burden of the primary lesion.

Toxicity is a crucial factor when adding RT to chemotherapy. CCRT significantly increased hematologic toxicities, including neutropenia, leukopenia, febrile neutropenia, and thrombocytopenia, both in all grades and in grades 3–4, compared to chemotherapy alone. Radiation-associated pneumonitis occurred in approximately 18% of the patients but it was generally mild. TE fistula occurred in approximately 5% of patients in the CCRT group, which was not significantly different from the 1.2% in the chemotherapy alone group. Overall, treatment-related AEs observed in this study were mild and manageable. These findings suggest that CCRT is generally tolerated and is clinically feasible in patients with advanced-stage ESCC. However, the risks and potential complications of RT, along with its benefits, should be carefully considered and discussed with individual patients.

A key strength of our study is its contribution to addressing the significant gap in the current knowledge regarding optimal treatment strategies to improve OS in patients with advanced-stage ESCC, especially when access to immunotherapy in addition to chemotherapy is limited. Furthermore, we employed statistical methods to adjust for the bias inherent to the nature of a retrospective study, including a multivariate Cox proportional hazards model to strengthen the validity of our results and provide a more accurate assessment of the benefits of CCRT. Additionally, our comprehensive subgroup analyses of OS times are essential for guiding physicians in tailoring treatment strategies and making informed decisions for individual patients.

This study has some limitations. The main limitation is its retrospective design, which lacks randomization and may introduce selection bias. To mitigate this issue, a multivariate Cox proportional hazards model was used to adjust for potential confounding variables. Second, the treatment regimens for the comparator group, who received chemotherapy alone, may not reflect the current standard of care, as immunotherapy is now included as a first-line treatment option. However, given the high cost of immunotherapy, many patients cannot afford it, making chemotherapy the standard practice in our setting. 

In conclusion, CCRT did not provide a benefit to all unselected patients with advanced-stage ESCC, and higher rates of adverse events were expected. Thus, adding concurrent RT is particularly considered for patients with stage IVA ESCC with good performance status, as it has been shown to improve OS, PFS, and ORRs compared to chemotherapy alone. Further prospective studies with larger sample sizes, especially those with distant metastases, should be conducted to assess the role of CCRT. Dysphagia scores and QoL should be evaluated as key concerns when considering CCRT, along with survival benefits.

## Figures and Tables

**Table 1:** Baseline characteristics.

	CCRT (n = 117)	Chemotherapy Alone (n = 79)
Median Age, years (IQR)	59.6 (54.7, 65.0)	60.3 (55.2, 67.4)
Age ≥ 65 years, n (%)	29 (24.8)	25 (31.6)
Sex, n (%)		
Male	107 (91.5)	75 (94.9)
Female	10 (8.5)	4 (5.1)
ECOG PS, n (%) *		
0	3 (2.6)	0 (0)
1	98 (83.8)	53 (67.1)
2	15 (12.8)	25 (31.6)
3	1 (0.9)	0 (0)
BMI, n (%)		
<18.5 kg/m^2^	66 (56.4)	45 (57.0)
18.5–22.9 kg/m^2^	35 (29.9)	25 (31.6)
23.0–24.9 kg/m^2^	9 (7.7)	4 (5.1)
≥25 kg/m^2^	7 (6.0)	5 (6.3)
Smoking, n (%)		
Current or former	101 (86.3)	74 (93.7)
Never	16 (13.7)	5 (6.3)
Alcohol drinking, n (%)		
Current or former	98 (83.8)	71 (89.9)
Never	19 (16.2)	8 (10.1)
Comorbidities, n (%)	54 (46.2)	34 (43.0)
Hypertension	32 (27.4)	19 (24.1)
Diabetes mellitus	11 (9.4)	5 (6.3)
Cirrhosis	9 (7.7)	7 (8.9)
Cerebrovascular disorder	8 (6.8)	5 (6.3)
COPD	6 (5.1)	1 (1.3)
Atherosclerotic heart disease	3 (2.6)	1 (1.3)
History of previous cancer, n (%)	6 (5.1)	8 (10.1)
Tumor Location, n (%)		
Cervical	19 (16.2)	3 (3.8)
Upper thoracic	21 (17.9)	14 (17.7)
Middle thoracic	48 (41.0)	40 (50.6)
Lower thoracic	27 (23.1)	19 (24.1)
Esophagogastric junction	2 (1.7)	3 (3.8)
Tumor differentiation, n (%)		
Well differentiated SCC	22 (18.8)	14 (17.7)
Moderately differentiated SCC	55 (47.0)	39 (49.4)
Poorly differentiated SCC	35 (29.9)	18 (22.8)
Missing	5 (4.3)	8 (10.1)
T stage, n (%) *		
T2	1 (0.9)	7 (8.9)
T3	39 (33.3)	41 (51.9)
T4	77 (65.8)	31 (39.2)
N stage, n (%)		
N0	6 (5.1)	4 (5.1)
N1	64 (54.7)	41 (51.9)
N2	29 (24.8)	19 (24.1)
N3	18 (15.4)	15 (19.0)
M stage, n (%) *		
M0	67 (57.3)	14 (17.7)
M1	50 (42.7)	65 (82.3)
Number of organ metastasis, n (%) *		
1	39 (33.3)	40 (50.6)
2	10 (8.5)	19 (24.1)
3	1 (0.9)	6 (7.6)
Organ metastasis, n (%)		
Lung *	15 (12.8)	25 (31.6)
Lymph node	28 (23.9)	29 (36.7)
Liver *	10 (8.5)	23 (29.1)
Bone	6 (5.1)	8 (10.1)
Others	3 (0.3)	9 (11.4)
Laboratory		
White blood cell count, per µL (IQR)	8930 (7200, 10,990)	9010 (7460, 11,530)
Hemoglobin, g/dL (IQR) *	11.4 (10.1, 12.7)	10.8 (9.7, 11.9)
Platelet count, per µL (IQR)	329,000 (256,000, 423,000)	338,000 (261,000, 433,000)
Creatinine, mg/dL (IQR)	0.8 (0.6, 0.9)	0.7 (0.6, 0.8)
CrCl, mL/min (IQR)	55.2 (46.8, 62.8)	52.9 (45.4, 62.1)
CrCl < 60 mL/min, n (%)	78 (66.7)	56 (71.8)
CrCl ≥ 60 mL/min, n (%)	39 (33.3)	22 (28.2)
Albumin, g/dL (SD) *	3.7 (0.5)	3.5 (0.5)

* Statistical significance between CCRT and CMT (*p*-value < 0.05). CCRT, concurrent chemoradiotherapy; IQR, interquartile range; ECOG, Eastern Cooperative Oncology Group; PS, performance status; BMI, body mass index; COPD, chronic obstructive pulmonary disease; SCC, squamous cell carcinoma; CrCl, creatinine clearance; and SD, standard deviation.

**Table 2:** Treatment information.

	CCRT (n = 117)	Chemotherapy Alone (n = 79)
Chemotherapy regimen, n (%) *		
Carboplatin plus 5-FU	52 (44.5)	56 (70.9)
Cisplatin plus 5-FU	59 (50.4)	18 (22.8)
Carboplatin plus paclitaxel	6 (5.1)	5 (6.3)
Treatment		
Dose of cisplatin, mg/m^2^ (IQR)	80 (80, 80)	80 (80, 80)
Dose of carboplatin, AUC (IQR)	4.5 (4.0, 5.0)	5.0 (4.0,5.0)
Dose of 5-FU, mg/m^2^/cycle (IQR)	4000 (3200, 4000)	4000 (3200, 4000)
Dose of paclitaxel, mg/m^2^ (IQR)	140 (140, 140)	140 (140, 175)
Any dose reduction, n (%)	52 (44.4)	36 (45.5)
One level	46 (39.3)	34 (43.0)
Two level	6 (5.1)	2 (2.5)
Number of chemotherapy cycles (IQR)	4 (2, 4)	3 (2, 5)
Concurrent RT	117 (100.0)	-
RT technique, n (%)		-
VMAT	84 (71.8)	-
IMRT	3 (2.6)	-
3D-CRT	30 (25.6)	-
Dose of RT, Gray (IQR)	50.4 (50.0, 54.0)	-
≥50 Gray, n (%)	94 (80.3)	-
<50 Gray, n (%)	23 (19.7)	-
Discontinuation, n (%)		
Complete treatment	44 (37.6)	15 (19.0)
Progressive disease	27 (23.1)	30 (38.0)
Death	18 (15.4)	27 (34.2)
Worsening ECOG PS	18 (15.4)	5 (6.3)
Patient preference	5 (4.3)	2 (2.5)
Loss of follow-up	3 (2.6)	0 (0)
Toxicity	2 (1.7)	0 (0)
Subsequent therapy, n (%)		
Second line treatment	26 (22.2)	21 (26.6)
Third line treatment	8 (6.8)	1 (1.3)
Fourth line treatment	1 (0.9)	0 (0)

* Statistical significance between CCRT and CMT (*p*-value < 0.05). CCRT: concurrent chemoradiotherapy; 5-FU: 5-fluorouracil; IQR: interquartile range; AUC: area under the curve; RT: radiotherapy; VMAT: Volumetric Modulated Arc Therapy; IMRT: Intensity-Modulated Radiation Therapy; 3D-CRT: three-dimension conformal radiotherapy; ECOG: Eastern Cooperative Oncology Group; and PS: performance status.

**Table 3:** Response rate.

	CCRT (n = 117)	Chemotherapy Alone (n = 79)	*p*-Value
Complete response, n (%)	0 (0)	0 (0)	
Partial response, n (%)	51 (43.6)	18 (22.8)	
Stable disease, n (%)	28 (23.9)	16 (20.3)	
Progressive disease, n (%)	20 (17.1)	19 (24.1)	
ORR per ITT, n (%)	51 (43.6)	18 (22.8)	0.003
ORR per assessable, n (%)	51 (51.5)	18 (34.0)	0.018

CCRT: concurrent chemoradiotherapy; ORR: objective response rate; and ITT: intention-to-treat.

**Table 4:** Safety.

AEs	CCRT (n = 116)		Chemotherapy Alone (n = 72)	
	All Grade n (%)	Grade 3–4 n (%)	All Grade n (%)	Grade 3–4 n (%)
Hematologic				
Neutropenia *^,^**	83 (71.6)	30 (25.9)	23 (31.9)	4 (5.6)
Leukopenia *^,^**	95 (81.9)	29 (25.0)	25 (34.7)	4 (5.6)
Thrombocytopenia *^,^**	58 (50.0)	22 (19.0)	13 (18.1)	7 (9.7)
Anemia	110 (94.8)	24 (20.7)	65 (90.3)	14 (19.4)
Febrile neutropenia *^,^**	18 (15.5)	18 (15.5)	2 (2.8)	2 (2.8)
Non-hematologic				
Nausea	20 (17.2)	1 (0.9)	6 (8.3)	0 (0.0)
Vomiting	18 (15.5)	1 (0.9)	6 (8.3)	0 (0.0)
Diarrhea	8 (6.9)	1 (0.9)	3 (4.2)	0 (0.0)
Elevated ALT	5 (4.3)	1 (0.9)	4 (5.6)	0 (0.0)
Fatigue	22 (19.0)	3 (2.6)	9 (12.5)	0 (0.0)
Radiation-associated				
Esophagitis	10 (8.6)	1 (0.9)	2 (2.8)	0 (0.0)
Pneumonitis *	21 (18.1)	0 (0.0)	4 (5.6)	0 (0.0)
Dermatitis *	25 (21.6)	5 (4.3)	3 (4.2)	0 (0.0)
Mucositis	17 (14.7)	1 (0.9)	6 (8.3)	0 (0.0)
Tracheoesophageal fistula	6 (5.2)	6 (5.2)	1 (1.4)	1 (1.4)

* Statistically significant difference of all grade AEs between CCRT and CMT (*p*-value < 0.05). ** Statistically significant difference of grade 3–4 AEs between CCRT and CMT (*p*-value < 0.05). AEs: adverse events; CCRT: concurrent chemoradiotherapy; and ALT: alanine transaminase.

## Data Availability

The datasets used and/or analyzed in the current study are available from the corresponding author upon reasonable request.

## References

[1] Sung H., Ferlay J., Siegel R.L., Laversanne M., Soerjomataram I., Jemal A., Bray F. (2021). Global Cancer Statistics 2020: GLOBOCAN Estimates of Incidence and Mortality Worldwide for 36 Cancers in 185 Countries. CA Cancer J. Clin..

[2] Lagergren J., Smyth E., Cunningham D., Lagergren P. (2017). Oesophageal cancer. Lancet.

[3] DaSilva L.L., Aguiar P.N., de Lima Lopes G. (2021). Immunotherapy for Advanced Esophageal Squamous Cell Carcinoma—Renewed Enthusiasm and a Lingering Challenge. JAMA Oncol..

[4] Tanaka T., Fujita H., Matono S., Nagano T., Nishimura K., Murata K., Shirouzu K., Suzuki G., Hayabuchi N., Yamana H. (2010). Outcomes of multimodality therapy for stage IVB esophageal cancer with distant organ metastasis (M1-Org). Dis. Esophagus.

[5] van Rossum P.S.N., Mohammad N.H., Vleggaar F.P., van Hillegersberg R. (2018). Treatment for unresectable or metastatic oesophageal cancer: Current evidence and trends. Nat. Rev. Gastroenterol. Hepatol..

[6] Mukkamalla S.K.R., Recio-Boiles A., Babiker H.M. (2023). Esophageal Cancer. StatPearls [Internet].

[7] Ku G.Y. (2017). Systemic therapy for esophageal cancer: Chemotherapy. Chin. Clin. Oncol..

[8] Grünberger B., Raderer M., Schmidinger M., Hejna M. (2007). Palliative Chemotherapy for Recurrent and Metastatic Esophageal Cancer. Anticancer. Res..

[9] Doki Y., Ajani J.A., Kato K., Xu J., Wyrwicz L., Motoyama S., Ogata T., Kawakami H., Hsu C.H., Adenis A. (2022). Nivolumab combination therapy in advanced esophageal squamous-cell carcinoma. N. Engl. J. Med..

[10] Puhr H.C., Prager G.W., Ilhan-Mutlu A. (2023). How we treat esophageal squamous cell carcinoma. ESMO Open.

[11] Sun J.M., Shen L., Shah M.A., Enzinger P., Adenis A., Doi T., Kojima T., Metges J.P., Li Z., Kim S.B. (2021). Pembrolizumab plus chemotherapy versus chemotherapy alone for first-line treatment of advanced oesophageal cancer (KEYNOTE-590): A randomised, placebo-controlled, phase 3 study. Lancet.

[12] Herskovic A., Martz K., Al-Sarraf M., Leichman L., Brindle J., Vaitkevicius V., Cooper J., Byhardt R., Davis L., Emami B. (1992). Combined chemotherapy and radiotherapy compared with radiotherapy alone in patients with cancer of the esophagus. N. Engl. J. Med..

[13] van Hagen P., Hulshof M.C.C.M., Van Lanschot J.J.B., Steyerberg E.W., Henegouwen M.V.B., Wijnhoven B.P.L., Richel D.J., Nieuwenhuijzen G.A.P., Hospers G.A.P., Bonenkamp J.J. (2012). Preoperative Chemoradiotherapy for Esophageal or Junctional Cancer. N. Engl. J. Med..

[14] Conroy T., Galais M.P., Raoul J.L., Bouché O., Gourgou-Bourgade S., Douillard J.Y., Etienne P.L., Boige V., Martel-Lafay I., Michel P. (2014). Definitive chemoradiotherapy with FOLFOX versus fluorouracil and cisplatin in patients with oesophageal cancer (PRODIGE5/ACCORD17): Final results of a randomised, phase 2/3 trial. Lancet Oncol..

[15] Li Q.Q., Liu M.Z., Hu Y.H., Liu H., He Z.Y., Lin H.X. (2010). Definitive concomitant chemoradiotherapy with docetaxel and cisplatin in squamous esophageal carcinoma. Dis. Esophagus.

[16] Li L.Q., Fu Q.G., Zhao W.D., Wang Y.D., Meng W.W., Su T.S. (2022). Chemoradiotherapy Versus Chemotherapy Alone for Advanced Esophageal Squamous Cell Carcinoma: The Role of Definitive Radiotherapy for Primary Tumor in the Metastatic Setting. Front. Oncol..

[17] Lyu J., Li T., Wang Q., Li F., Diao P., Liu L., Li C., Lang J. (2018). Outcomes of concurrent chemoradiotherapy versus chemotherapy alone for stage IV esophageal squamous cell carcinoma: A retrospective controlled study. Radiat. Oncol..

[18] Ajani J.A., Barthel J.S., Bentrem D.J., D’Amico T.A., Das P., Denlinger C.S., Fuchs C.S., Gerdes H., Glasgow R.E., Hayman J.A. (2011). Esophageal and Esophagogastric Junction Cancers. J. Natl. Compr. Canc Netw..

[19] Muro K., Lordick F., Tsushima T., Pentheroudakis G., Baba E., Lu Z., Cho B.C., Nor I.M., Ng M., Chen L.T. (2019). Pan-Asian adapted ESMO Clinical Practice Guidelines for the management of patients with metastatic oesophageal cancer: A JSMO–ESMO initiative endorsed by CSCO, KSMO, MOS, SSO and TOS. Ann. Oncol..

[20] McKernan M., McMillan D.C., Anderson J.R., Angerson W.J., Stuart R.C. (2008). The relationship between quality of life (EORTC QLQ-C30) and survival in patients with gastro-oesophageal cancer. Br. J. Cancer.

[21] Wonglhow J., Wetwittayakhlang P., Sunpaweravong P., Sathitruangsak C., Dechaphunkul A. (2024). Comparing the Efficacy of Carboplatin plus 5-Fluorouracil, Cisplatin plus 5-Fluorouracil, and Best Supportive Care for Advanced Esophageal Squamous Cell Carcinoma: A Propensity Score Analysis from a Tertiary Hospital in Southern Thailand. J. Clin. Med..

[22] Lancellotta V., Cellini F., Fionda B., De Sanctis V., Vidali C., Fusco V., Barbera F., Gambacorta M.A., Corvò R., Magrini S.M. (2020). The role of palliative interventional radiotherapy (brachytherapy) in esophageal cancer: An AIRO (Italian Association of Radiotherapy and Clinical Oncology) systematic review focused on dysphagia-free survival. Brachytherapy.

[23] Lee J., Im Y.H., Cho E.Y., Hong Y.S., Lee H.R., Kim H.S., Kim M.J., Kim K., Kang W.K., Park K. (2008). A phase II study of capecitabine and cisplatin (XP) as first-line chemotherapy in patients with advanced esophageal squamous cell carcinoma. Cancer Chemother. Pharmacol..

[24] Zhang X.D., Shen L., Li J., Li Y., Zhang X.T., Jin M.L. (2007). Prospective non-randomized study of chemotherapy combined with radiotherapy versus chemotherapy alone in patients with metastatic or relapsed esophageal squamous cell carcinoma. Zhonghua Zhong Liu Za Zhi.

[25] Obermannová R., Alsina M., Cervantes A., Leong T., Lordick F., Nilsson M., van Grieken N.C.T., Vogel A., Smyth E.C. (2022). Oesophageal cancer: ESMO Clinical Practice Guideline for diagnosis, treatment and follow-up. Ann. Oncol..

[26] Xu J., Lu D., Zhang L., Li J., Sun G. (2019). Palliative resection or radiation of primary tumor prolonged survival for metastatic esophageal cancer. Cancer Med..

[27] Li T., Lv J., Li F., Diao P., Wang J., Li C., Liang L., Sun L. (2016). Prospective Randomized Phase 2 Study of Concurrent Chemoradiation Therapy (CCRT) Versus Chemotherapy Alone in Stage IV Esophageal Squamous Cell Carcinoma (ESCC). Int. J. Radiat. Oncol. Biol. Phys..

[28] Chen Y., Cheng X., Song H., Wu A.J., Ku G.Y., Lee P., Slingerland M., Koyanagi K., Ke S., Qiu H. (2019). Outcomes of concurrent chemoradiotherapy versus chemotherapy alone for esophageal squamous cell cancer patients presenting with oligometastases. J. Thorac. Dis..

[29] Guttmann D.M., Mitra N., Bekelman J., Metz J.M., Plastaras J., Feng W., Swisher-McClure S. (2017). Improved Overall Survival with Aggressive Primary Tumor Radiotherapy for Patients with Metastatic Esophageal Cancer. J. Thorac. Oncol..

[30] Moreno A.C., Zhang N., Giordano S., Komaki R.U., Liao Z., Nguyen Q.N., Hofstetter W., Murphy M.B., Lin S.H. (2017). Comparative Effectiveness of Chemotherapy Alone Versus Chemotherapy and Radiation Therapy for Patients with Stage IV Esophageal Cancer. Int. J. Radiat. Oncol. Biol. Phys..

[31] Wada Y., Anbai A., Takagi N., Kumagai S., Okuyama E., Nanjo H., Sato Y., Motoyama S., Hashimoto M. (2020). Outcomes of Definitive Chemoradiotherapy for Stage IVa (T4b vs. N4) Esophageal Squamous Cell Carcinoma Based on the Japanese Classification System: A Retrospective Single-Center Study. Cancers.

[32] Ishida K. (2004). Phase II Study of Cisplatin and 5-Fluorouracil with Concurrent Radiotherapy in Advanced Squamous Cell Carcinoma of the Esophagus: A Japan Esophageal Oncology Group (JEOG)/Japan Clinical Oncology Group Trial (JCOG9516). Japan J. Clin. Oncol..

[33] Chen B., Deng M., Yang C., Dragomir M.P., Zhao L., Bai K., Xi M., Hu Y., Zhu Y., Li Q. (2021). High incidence of esophageal fistula on patients with clinical T4b esophageal squamous cell carcinoma who received chemoradiotherapy: A retrospective analysis. Radiother. Oncol..

[34] Makino T., Yamasaki M., Tanaka K., Miyazaki Y., Takahashi T., Kurokawa Y., Motoori M., Kimura Y., Nakajima K., Mori M. (2019). Treatment and clinical outcome of clinical T4 esophageal cancer: A systematic review. Ann. Gastroent Surg..

[35] Bleiberg H., Conroy T., Paillot B., Lacave A.J., Blijham G., Jacob J.H., Bedenne L., Namer M., De Besi P., Gay F. (1997). Randomised phase II study of cisplatin and 5-fluorouracil (5-FU) versus cisplatin alone in advanced squamous cell oesophageal cancer. Eur. J. Cancer.

